# Phosphorus *K*_4_ Crystal: A New Stable Allotrope

**DOI:** 10.1038/srep37528

**Published:** 2016-11-18

**Authors:** Jie Liu, Shunhong Zhang, Yaguang Guo, Qian Wang

**Affiliations:** 1 Center for Applied Physics and Technology, College of Engineering, Peking University; Key Laboratory of High Energy Density Physics Simulation, Ministry of Education, Beijing 100871, China; 2 Department of Materials Science and Engineering, College of Engineering, Peking University, Beijing 100871, China; 3 Collaborative Innovation Center of IFSA (CICIFSA), Shanghai Jiao Tong University, Shanghai 200240, China

## Abstract

The intriguing properties of phosphorene motivate scientists to further explore the structures and properties of phosphorus materials. Here, we report a new allotrope named *K*_4_ phosphorus composed of three-coordinated phosphorus atoms in non-layered structure which is not only dynamically and mechanically stable, but also possesses thermal stability comparable to that of the orthorhombic black phosphorus (*A*17). Due to its unique configuration, *K*_4_ phosphorus exhibits exceptional properties: it possesses a band gap of 1.54 eV which is much larger than that of black phosphorus (0.30 eV), and it is stiffer than black phosphorus. The band gap of the newly predicted phase can be effectively tuned by appling hydrostastic pressure. In addition, *K*_4_ phosphorus exibits a good light absorption in visible and near ultraviolet region. These findings add additional features to the phosphorus family with new potential applications in nanoelectronics and nanomechanics.

Phosphorus is a common material for industrialized production of fertilizers, organophosphorus compounds, matches, and so on. Because of its high chemical reactivity and strong toxicity, this material has received little academic attention until recently^1^. In 2014 phosphorus was brought to spotlight by successfully fabricating the field-effect transistors (FETs) constructed using the exfoliated black phosphorus and finding their outstanding performance by two independent groups^2^,^3^, which was exactly 100 years after the discovery of black phosphorus^4^. Recently, monolayer black phosphorus (phosphorene), as a new member of two dimensional (2D) materials family, has attracted tremendous attention to explore the structural varieties^5^, electronic properties^6^, unique mechanical features^7^, and promising applications in electronics^8^ and gas sensors^9^.

Like carbon and boron, phosphorus displays fascinating structural variability. The stable allotropes of phosphorus at ambient conditions can be classified into three major categories: white phosphorus^10^, orthorhombic black phosphorus^4^, and various forms of red phosphorus^11^. White phosphorus is highly reactive with air and forms three crystal structures including α-, β-, and γ-P_4_^12^. Black phosphorus possesses an orthorhombic structure (*A*17, space group: *Cmca*) at ambient conditions and transforms to rhombohedral structure (*A*7, space group: *R*
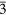
*m*) at around 5 GPa^12^. While the *A*7 structure transforms to an α-Po-type simple cubic three dimensional (3D) structure (space group: *Pm3m*) at a higher pressure of about 10 GPa^10^. Amorphous and crystalline forms of red phosphorus can be evolved from white phosphorus by heating it to higher than 20 °C^13^. The various forms of red phosphorus are based on tubular units of five- and six-membered rings^12^. Although a number of the allotropes of phosphorus have already been experimentally characterized or theoretically predicted^4^,^12^,^14^ the discovery of new phases of phosphorus has attracted continued attention^15^,^16^. Since the most stable phase of phosphorus is the three-coordinated *layered* black phosphorus, an interesting question then raises: can we find a stable 3D *non-layered* allotrope of phosphorus at ambient conditions which is also composed of only three-coordinated atoms?

Inspired by the unique geometry of *K*_4_ structure where the coordination number of each atom is three, we explore the stability and properties of phosphorus in *K*_4_ crystal structure. In fact, since the identification of *K*_4_ geometry in mathematics in 2008^17^, design and synthesis of pristine elemental substances in *K*_4_ structure have been an attractive scientific topic. The nitrogen *K*_4_ crystal named cg-N (cubic gauche) was synthesized from molecular nitrogen under pressure above 110 GPa using a laser-heated diamond cell^18^. The coordination polymers and metal-organic frameworks having the topology of *K*_4_ crystal were reported in 1990s^19^. A *K*_4_ crystal carbon was proposed to be a possible metallic allotrope of carbon in 2009^20^. Unfortunately, a latter work suggested that *K*_4_ carbon is dynamically unstable^21^. In 2010, Dai *et al*. proposed a boron *K*_4_ crystal that is stable under ambient pressure^22^. All these progresses, especially experimental realization of high pressure polymeric nitrogen in *K*_4_ structure, make the exploration of *K*_4_ phosphorus structure very promising.

In this work, the dynamical, thermal, and mechanical stabilities of *K*_4_ phosphorus structure are confirmed, and its electronic, mechanical, and optical properties are studied based on a series of state-of-the-art calculations. The phonon vibrational modes at the first Brillouin zone center are also simulated to aid future experimental identification of the new phase of phosphorus from Raman and infrared (IR) spectroscopy.

## Results and Discussion

### Phosphorus *K*_4_ structure

**Figure 1:**
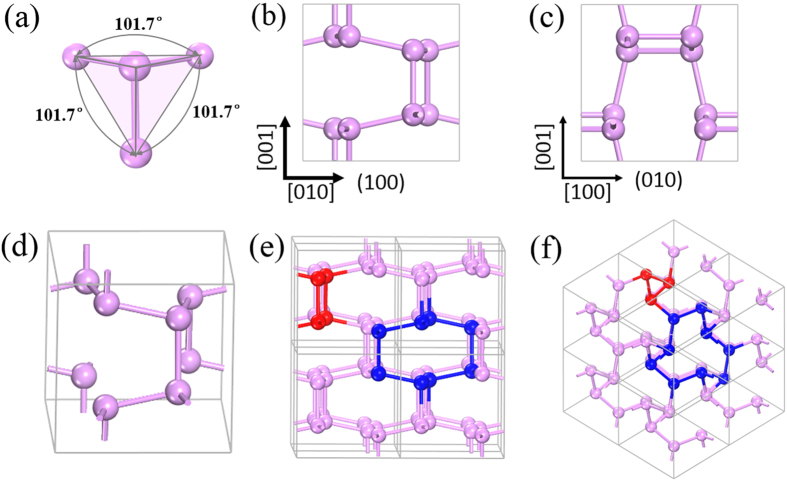
Optimized crystal structure of *K*_4_ phosphorus. (**a**) Building block of *K*_4_ phosphorus. (**b**)–(**c**) Crystal structure of *K*_4_ phosphorus viewed from the [100] and [010] directions. (**d**) Perspective view of the conventional unit cell of *K*_4_ phosphorus. (**e**) and (**f**) Two different perspective views of a 2 × 2 × 2 supercell to display the charity.

Different from boron *K*_4_ crystal, where each boron atom is *sp*^*2*^-hybridized, for the stable phosphorus allotropes at ambient conditions, due to the electron lone pair, each phosphorus atom is actually *sp*^*3*^-hybridized which displays a tetrahedral bonding character^6^. To remain this bonding character, we conceive the idea of building a phosphorus *K*_4_ structure using a building block composed of four phosphorus atoms in a tetrahedron configuration that is displayed in Fig. 1(a). In the building block, the central phosphorus atom is *sp*^3^-hybridized, and connects its three neighboring atoms, thus remaining the tetrahedral bonding character of phosphorus at ambient conditions. With equal bond lengths and bond angles, this structural unit is used to build the high symmetric *K*_4_ crystal.

As shown in Fig. 1(c) and (d), the optimized structure of phosphorus *K*_4_ is body-centered cubic with 8 atoms located at the 8a (0.206, 0.206, 0.206) Wyckoff position in the conventional unit cell. Compared to *K*_*4*_ boron, *K*_*4*_ phosphorus has a reduced symmetry with the space group symmetry of T5 (I2_1_3, No. 199). The lattice parameters are optimized using both PBE and GGA-D2 functionals, and the results are presented in Table 1. The lattice parameters of black phosphorus are also calculated using the same level of theory and compared with the experimental data. We can see that *K*_4_ phosphorus is slightly denser than the layered phosphorus structure of *A*17. However, the mass density difference is much smaller than that between diamond and graphite. This is because the electron-rich nature and the resultant Coulomb repulsion hinder phosphorus to form ultra-dense phases like diamond. As compared to black phosphorus, *K*_4_ phosphorus has a higher symmetry, leading to an isotropic network. In the unit cell of *K*_4_ phosphorus, only one kind of P-P bond exists and all bond angles are equivalent with a bond length of 2.24 Å/2.25 Å and a bond angle of 101.7°/102.2° at the GGA-D2/PBE level. In addition, as shown in Fig. 1(e) and (f), the three-coordinated phosphorous network displays an intriguing chirality that can be seen from the spiral square-octagon and triangle-nonagon polygons pairs.

### Energetic stability

To investigate the energetic stability of *K*_4_ phosphorus, total energy calculations are performed by using both the PBE and GGA-D2 functionals, respectively. For comparison, calculations are also carried out for black phosphorus. The results are listed in Table 1. The cohesive energy of *K*_4_ phosphorus is found to be higher than that of black phosphorus by 0.02/0.04 eV/atom at the PBE/GGA-D2 functional, showing that *K*_4_ phosphorus is thermodynamically metastable as compared to the most stable form of phosphorous allotropes, black phosphorus^12^. To further compare the relative stability of *K*_4_ phosphorus with other phosphorus allotropes, the total energies of some other allotropes are also calculated at PBE/GGA-D2 level, and found that *K*_4_ phosphorus is 0.07/0.01 eV/atom lower in energy than the simple cubic structure, and 0.08/0.14 eV/atom lower in energy than the two structures of white phosphorus (β-P_4_, γ-P_4_)^13^, indicating that the *K*_4_ phosphorus phase is energetically more stable than these well-known structures. To understand the reason why *K*_4_ phosphorus is relatively stable, we investigated its atomic configuration. We note that in the three-coordinated phosphorus *K*_4_ structure each bond angle is 101.7°, as shown in Fig. 1(a), close to that of 101.6° in the P_4_H_6_ molecule that was found to be the most energetically favorable configuration among those of the three-coordinated phosphorus atoms^5^. Therefore, the favorable geometry results in a good energetic stability of the *K*_4_ structure.

To search for 3D *non-layered* phosphorus allotropes with good energetic stability at ambient conditions, we have screened many possible 3-coordintated networks. Since the phosphorus *K*_4_ structure has the **srs** topology, by using the network topology approach based on the RCSR database^23^,^24^, many candidate structures for 3D *non-layered* phosphorus with different network topologies can be obtained. Due to the limited computational resources, we only consider the so called uninodal (with only one type of vertex) structures. Actually there are 78 uninodal 3-coordinated nets in the RCSR database. Among them, there are 47 nets with too short non-bonded distance, and there are 1 net with the bond angles seriously deviated from that of P_4_H_6_ molecule (101.6°)^5^. So these 48 nets should be excluded. However, not all the 30 rest nets are suitable for forming 3D *non-layered* phosphorus structures. For example, the optimized structures of **etb** and **utp** configurations become 5-coordinated, and the optimized structure of **utg** configuration turns into phosphorus chains. Finally, only 14 nets including the **srs** net (*K*_4_ phosphorus) are found to be the suitable candidate structures for 3D *non-layered* phosphorus allotropes in which every phosphorus atom retains the *sp*^3^ hybridization character of tetra-phosphorus. The cohesive energy of these hypothetical phosphorus structures are calculated by using the PBE and GGA-D2 functionals, respectively. As listed in Table 2, we find that *K*_4_ phosphorus is energetically most stable among the studied configurations.

### Dynamic stability

**Figure 2:**
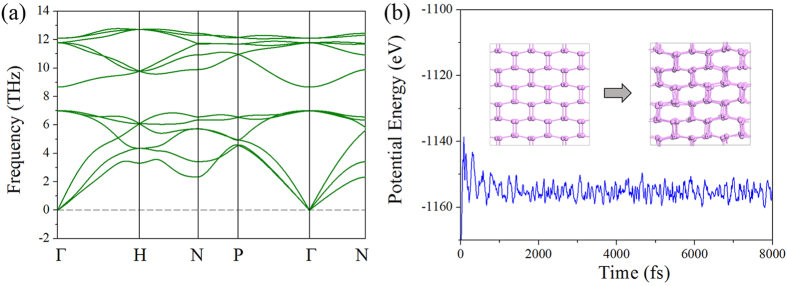
Structure stability of *K*_4_ phosphorus. (**a**) Vibrational band structure of *K*_*4*_ phosphorus. (**b**) Total potential energy fluctuation of *K*_*4*_ phosphorus during AIMD simulation at 1000 K. The inset shows the atomic configurations (3 × 3 × 3 supercell) at the beginning and end of AIMD simulations at 1000 K.

To examine the dynamic stability of *K*_4_ phosphorus, the lattice dynamics is studied by calculating its phonon dispersion using linear response method within density functional perturbation theory^25^, where the force-constant matrix is calculated through differentiation of the Hellmann-Feynman forces on atoms with respect to the ionic coordinates. As shown in Fig. 2(a), the absence of imaginary modes in the whole Brillouin zone confirms that *K*_4_ phosphorus is dynamically stable. The primitive cell of *K*_4_ phosphorus contains four atoms, leading to nine optical and three acoustic branches. The optical branches can be classified into two groups with a frequency gap between them. The low energy group consisting of three optical branches is predominantly bond-bending type in character, while the high energy group consisting of six optical branches is bond-stretching type. All the three acoustic branches of *K*_4_ phosphorus are linearly dispersed near the Γ point in different directions, confirming the relatively strong covalent bonds between the phosphorus atoms along these directions, while in the structure of black phosphorus the dispersion relation of the TA_z_ modes near the Γ point in the [100] and [010] directions^26^ is almost in quadratic form that is contributed by the weak interlayer interactions.

### Thermal stability

The thermal stability of *K*_4_ phosphorus is examined by performing *ab initio* molecular dynamics (AIMD) simulations at 300 K with a large supercell (3 × 3 × 3). We find that no structure reconstruction occurs after heating for 8 *ps* with a time step of 1 *fs*, and the total potential energy remains almost constant during the simulation. These results suggest that the new structure is thermally stable at room temperature. The heat bath is then further elevated to 1000 K. As shown in the snapshot of atomic configuration of *K*_4_ phosphorus at the end of AIMD simulations at 1000 K (see Fig. 2(b)), after heating for 8 *ps*, no obvious distortion in the structure appears, and the fluctuation in total potential energy still remains almost unchanged. This implies that *K*_4_ phosphorus can withstand temperatures up to 1000 K, and this phosphorus phase is separated by high energy barriers from other local minima on the potential energy surface (PES) of elemental phosphorus.

However, it is worthy to note that the temperature for evaluating the thermal stability of a crystal structure may be overestimated by AIMD simulations due to using Canonical ensemble (NVT) during the simulations. To further investigate the thermal stability of *K*_4_ phosphorus, the AIMD simulations are also carried out for the experimentally synthesized *A*17 structure for comparison. As shown in Fig. S1, the A17 structure can withstand the high temperature of 1000 K without any obvious structural reconstruction. However, when temperature of the heat bath is further increased to 1200 K, both the geometries of the *K*_4_ and *A*17 phases are destroyed. The results reveal that *K*_4_ phosphorus is thermally as stable as the *A*17 phase.

### Mechanical stability and properties

To guarantee the positive definiteness of strain energy upon lattice distortion, the mechanical stability of *K*_4_ phosphorus is examined. In the linear elastic range the elastic constant tensor forms a 6 × 6 matrix with 21 independent components. For a simple cubic lattice, only *C*_11_, *C*_12_ and *C*_44_ are independent. The linear elastic constants of a mechanically stable 3D cubic lattice have to obey the Born-Huang criteria: *C*_11_ > 0, *C*_44_ > 0, *C*_11_ > |*C*_12_|, and (*C*_11_ + 2*C*_12_) > 0^27^. The elastic constants of the phosphorus *K*_4_ crystal are derived from the strain-stress relationship by using the finite distortion method^28^ implemented in VASP. All the elastic constants calculated with both the PBE and the vdW-corrected GGA-D2 functionals are listed in Table 3. These constants obey all of the Born-Huang criteria for simple cubic lattices, implying that the new structure is mechanically stable. For comparison, the calculations are also performed for *A*17, and the results are given in Table 3 as well.

The *C*_11_, *C*_22_, and *C*_33_ (*C*_11_ = *C*_22_ = *C*_33_) elastic constants of *K*_4_ phosphorus directly relate to sound propagation along the crystallographic *a*, *b*, and *c* axes, respectively, and reflect the stiffness to the uniaxial strains along these directions. The calculated value of *C*_11_ is 223.6/232.2 GPa at the PBE/GGA-D2 level. While for the orthorhombic black phosphorus, *C*_22_ is significantly larger than *C*_11_ and *C*_33_ due to its structural anisotropy^29^, but it is smaller than the *C*_11_ elastic constant of *K*_4_ phosphorus, indicating that the *K*_4_ structure is stiffer than black phosphorus for strains along the *a, b*, and *c* axis. The benefit from the high stiffness is that the *K*_4_ structure could avoid the sliding observed in the layered phosphorus structures under a shear stress. The resistance of sliding could make *K*_4_ phosphorus more suitable for nano-mechanical applications. The single crystal bulk moduli of the phosphorus *K*_4_ crystal and black phosphorus are calculated according to the formula of bulk modulus represented by single-crystal elastic constants^27^. The result for black phosphorus is in good agreement with the value estimated from fitting the energy-volume relationship^30^.

### Electronic properties

**Figure 3:**
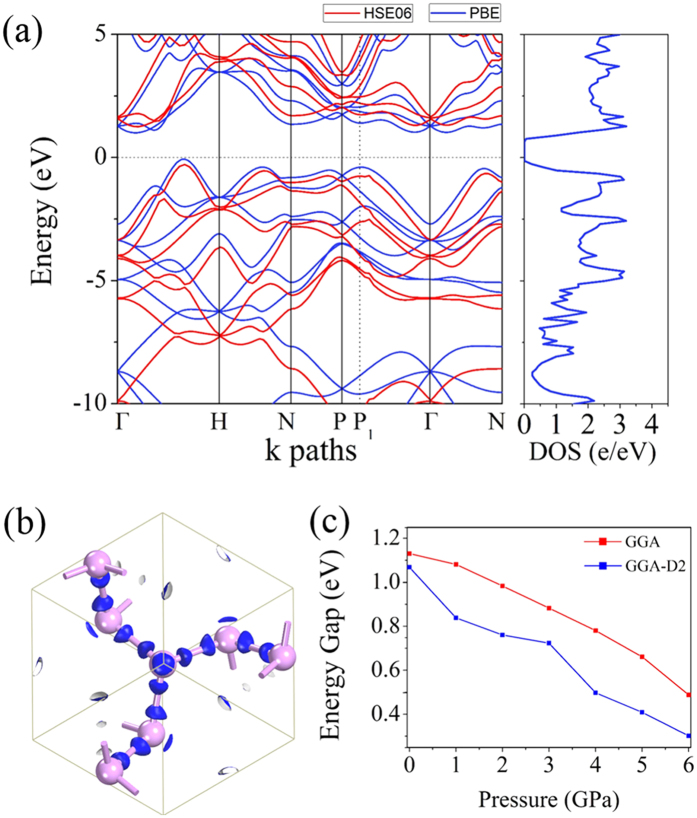
Electronic properties. (**a**) Electronic band structure and total DOS of *K*_4_ phosphorus using the PBE (blue lines) and the HSE06 hybrid functionals (red lines). (**b**) Isosurces (0.76 e/Å^3^) of the total valence electron density calculated using the PBE functionals. (**c**) Variation of band gap versus pressure calculated with the PBE and GGA-D2 functionals, respectively.

We calculate the electronic band structure and corresponding total density of states (DOS) of *K*_4_ phosphorus to study its electronic properties. The results are displayed in Fig. 3(a). At the PBE level, *K*_4_ phosphorus is predicted to be an indirect band gap semiconductor with a band gap of 1.07 eV as the valence band maximum (VBM) and the conduction band minimum (CBM) lie at the different points along the Γ-H path. It is well-known that the PBE funtional underestimates the fundamental band gaps of semiconductors, thus the band gap of *K*_4_ phosphorus is corrected by calculations using the more accurate HSE06 functional. As shown in Fig. 3(a). although both of the functionals give very similar band dispersions, the band gap of *K*_4_ phosphorus calculated with HSE06 functional is increased to 1.54 eV. Figure 3(b) shows the isosurfaces of the toatal valence electron density of *K*_4_ phosphorus. As mentioned above, the phosphorus atoms are *sp*^*3*^ hybridized due to the three P-P covelent bonds and a lone electron pair, thus the valence electrons mainly distributed along the directions of the P-P bonds and the lone electron pair.

To investigate how the energy band gap of *K*_4_ phosphorus changes with the applied hydrostatic pressure, we calculate the band gap as a function of the pressure, and plot the results in Fig. 3(c), which shows that the band gap of the *K*_4_ phase decreases with volume compression under hydrostastic pressure. At the GGA level, the band gap decreases almost linealy from 1.13 eV to 0.49 eV as pressure increses from zero to 6 GPa. While at the GGA-D2 level, the band gap decreases from 1.07 eV to 0.30 eV within the same pressure range.

### Optical properties

**Figure 4:**
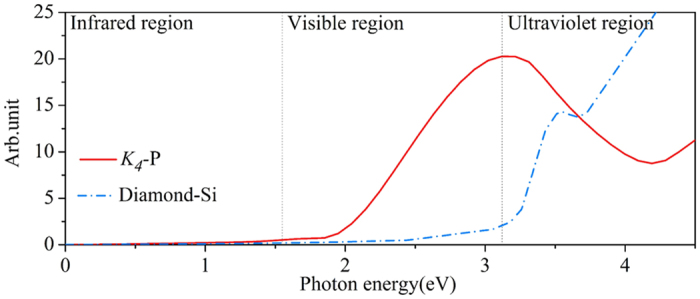
Optical absorption spectra. Imaginary part of dielectric function of *K*_4_ phosphorus and diamond silicon calculated at the HSE06 level.

We next explore the potential applications of *K*_4_ phosphorus in optoelectronics. The imaginary part of dielectric function of *K*_4_ phosphorus, which is directly related to its optical absorbance, is calculated at the HSE06 level. For comparison, calculation for diamond silicon is also carried out by using the same approach. According to photon energy, the spectrum is divided into three parts, namely the infrared, visible, and ultraviolet regions, respectively. As shown in Fig. 4, the imaginary part of the dielectric function of *K*_4_ phosphorus reaches the maximum at 3.18 eV, and follows by a minimum at 4.21 eV. According to the calculated results, *K*_4_ phosphorus exhibits much stronger optical absorption than diamond silicon in the visible range. The absorption spectrum of *K*_4_ phosphorus is also higher than that of diamond silicon from 3.12 to 3.66 eV in the near ultraviolet region. Currently, diamond silicon is still the leading material of solar cells^31^. It has an indirect band gap of 1.1 eV and a large direct gap of 3.3 eV, making it inefficient for sunlight absorption^32^. For *K*_4_ phosphorus, although it is also an indirect band gap semiconductor, it possesses a direct band gap of 2.4 eV at P_1_ point in the Brillouin zone (see Fig. 3(a)), which just lies in the middle part of the spectral range of visible light. As compared with diamond silicon, the smaller direct band gap of *K*_4_ phosphorus makes it a better solar absorber. Based on above analysis, we conclude that *K*_4_ phosphorus exhibits strong optical absorption in the visible and near ultraviolet region, making it a promising candidate for photovoltaics.

To provide a possible way to experimentally identify the *K*_4_ phase of phosphorus from other phosphorus allotropes using Raman and infrared (IR) spectroscopy, we simulate the vibration properties of the *K*_4_ structure at the Γ point. In order to verify the reliability of our calculations, we first perform the calculations for the *A*17 phase. From the analysis of the D_*2h*_ point group, the zone-center optical phonon modes of *A*17 can be classified into


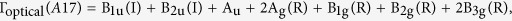


where A_g_, B_1g_, B_2g,_ and B_3g_ are Raman-active modes (marked with “R”), B_1u_ and B_2u_ are infrared-active modes (marked with “I”), and the A_u_ mode is silent. The frequencies of the Raman-active modes calculated at GGA-D2 level are 185.2, 219.8, 347.6, 410.9, 426.2, and 454.1 cm^−1^. These calculated results of Raman-active modes are in good agreement with earlier studies calculated using the same functional^29^.

The phosphorus *K*_4_ crystal belongs to T point group and possesses 4 irreducible representations, namely A, E, E^*^, and T, respectively. The symmetries of its optical phonons at the zone center can be represented by the irreducible representations of T point group:


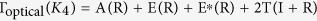


**Figure 5:**
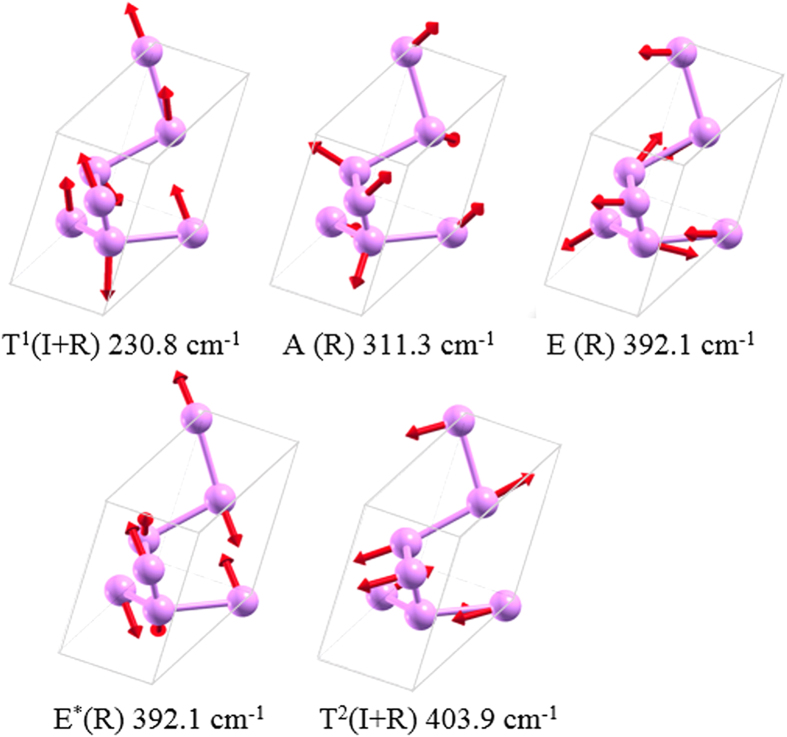
Vibrational modes. Snapshots of Raman-active and infrared-active modes for the *K*_4_ phase of phosphorus in the primitive cell.

All the optical phonons are Raman active, and the T^1^ and T^2^ modes are also infrared active. The frequencies of the five Raman-active modes are 230.8 (T^1^ mode), 311.3 (A mode), 392.1 (E mode), 392.1(E^*^ mode), and 403.9 (T^2^ mode) cm^−1^, respectively. The eigenvectors of the vibrational modes, as calculated at the GGA-D2 level, are illustrated in Fig. 5. These simulated Raman vibration results would be helpful to identify the *K*_4_ phosphorus structure in experiment.

The satisfactory stability of *K*_4_ phosphorus due to its ideal configuration of three-coordinated phosphorus atoms implies the possibility of the existence of this phase. Although it might be challenging to synthesize the *K*_4_ crystal, there are some relevant experimental findings that are supportive of our predication. As mentioned above, the counterpart of *K*_4_ phosphorus, the nitrogen *K*_4_ crystal, was theoretically predicted *via* first principles calculations in 1992^33^, and subsequently synthesized in 2004 through the polymerization of the molecular form of nitrogen at temperature above 2000 K and pressures above 110 GPa^18^. Similarly, the polymerization of the molecular form of phosphorus (tetrahedral P_4_ molecules) was reported by Katayama *et al*.^34^, where the transformation from molecular phosphorus to polymeric phosphorus occurred *via* thermal collision of the tetrahedral units of P_4_ molecules, and then a 3D network of three-coordinated phosphorus was formed. During such process, the experimental conditions, such as elevated temperature and environment of the tetramers play a decisive role^35^. Therefore, we conclude that the phosphorus *K*_4_ crystal could be synthesized through polymerization of molecular form of phosphorus at certain conditions as is the case with cg-N^18^.

## Summary

In summary, based on first principles calculations, we predict a 3D stable phosphorus *K*_*4*_ phase. We show that the *K*_4_ phosphorus structure is energetically metastable. The energy difference between the *K*_4_ phase and black phosphorus is very small (0.04 eV/atom), similar to that between diamond and graphite. Compared to other stable phosphorus allotropes at ambient conditions, such as layered phosphorus structure (*A*17 and violet phosphorus) and the molecular forms of phosphorus (modifications of white phosphorus), *K*_4_ phosphorus is a non-layered 3D phase with covalent bonds in all three dimensions. We demonstrate that *K*_4_ phosphorus possesses special properties different from those of black phosphorus. *K*_4_ phosphorus is stiffer than black phosphorus, and has a much larger band gap of 1.54 eV, which can be effectively tuned by appling hydrostastic pressure. *K*_4_ phosphorus exhibits much better optical absorbance than diamond silicon from 1.50 to 3.66 eV, which may have potential applications in optoelectronics. In addition, all optical phonons of *K*_4_ phosphorus at the first Brillouin zone center are Raman active and the T^1^ and T^2^ modes are also infrared active, which can be used to identify this new allotrope of phosphorus experimentally in the furure. We hope that the present theoretical study would shed new lights on discovery of novel phosphorous materials and motivate experimental efforts in this direction.

## Methods

First principles calculations are performed based on density functional theory (DFT) and the projector augmented wave (PAW) method^36^ as implemented in the Vienna *Ab initio* Simulation Package (VASP)^37^. The electronic exchange-correlation interaction is incorporated in Perdew-Burke-Enzerhof (PBE) functional^38^. The plane-wave cutoff energy for wave function is set to 500 eV. Lattice parameters and atomic positions are allowed to fully relax within the conjugate gradient algorithm. Since van der Waals (vdW) interactions are important for phosphorus allotropes^12^, the GGA-D2 functional^39^ with long-range dispersion is used for the refinement of geometry in addition to the PBE functional. The Heyd-Scuseria-Ernzerhof (HSE06)^40^ hybrid functional is then used for the high accuracy of electronic structure calculations. For structure optimization, the convergence thresholds are set at 10^−4^ eV and 10^−3^ eV/Å for total energy and force component, respectively. Monkhorst-Pack k-mesh^41^ of 9 × 9 × 9 is adopted to represent the first Brillouin zone. Thermal stability is studied using the Canonical ensemble (NVT) *ab initio* molecular dynamics (AIMD) simulations with temperature controlled by Nosé thermostat^42^. Phonon properties are calculated using the linear response method within density functional perturbation theory^25^ as implemented in the Phonopy code^43^. Elastic constants are determined by the finite distortion formalism^27^.

## Additional Information

**How to cite this article**: Liu, J. *et al*. Phosphorus *K*_4_ Crystal: A New Stable Allotrope. *Sci. Rep*. **6**, 37528; doi: 10.1038/srep37528 (2016).

**Publisher’s note:** Springer Nature remains neutral with regard to jurisdictional claims in published maps and institutional affiliations.

## Supplementary Material

Supplementary Information

## Figures and Tables

**Table 1:** Calculated lattice parameters (*a*, *b*, and *c* in Å), volume (*V*_*0*_ in Å^3^/atom), relative energies with respect to that of black phosphorus (Δ*E* in eV/atom), and energy band gaps (*E*_g_ in eV).

	*a*	*b*	*c*	*V*_*0*_	Δ*E*	*E*_g_
*K*_*4*_	5.37^a^	5.37	5.37	19.36^a^	0.02^a^	1.13^a^
	5.32^b^	5.32	5.32	18.82^a^	0.04^b^	1.54^d^ (1.07^b^)
*A*17	3.28^a^	11.22^a^	4.54^a^	20.89^a^	—	—
	3.32^b^	10.43^b^	4.41^b^	19.09^a^	—	0.36^d^ (0.08^a^)
	3.3133^c^	10.473^c^	4.374^c^	18.97^a^	—	0.335^c^

^a^Our calculated results at the PBE level.

^b^Our calculated results at the GGA-D2 level.

^c^Experimental data^12^.

^d^Our calculated results at the HES06 level.

**Table 2:** Relative energies of hypothetical phosphorus structures with respect to the cohesive energy of *K*_4_ phosphorus (Δ*E* in eV/atom).

	srs	acs-g	bcu-f	eta	etd	etf	pbg
Δ*E*^a^	0.000	0.085	0.216	0.241	0.141	0.235	0.116
Δ*E*^b^	0.000	0.145	0.273	0.239	0.197	0.254	0.132
	**pbp**	**pcu-g**	**pcu-h**	**ths**	**rhr-a**	**uct**	**uto**
Δ*E*^a^	0.608	0.563	0.023	0.017	0.409	0.308	0.293
Δ*E*^b^	0.721	0.663	0.031	0.096	0.517	0.365	0.354

^a^Calculated results at the PBE level.

^b^Calculated results at the GGA-D2 level.

**Table 3:** Calculated elastic constants (*C*_*ij*_ in GPa) and bulk moduli (B in GPa) of *K*_4_ phosphorus and orthorhombic black phosphorus (*A*17).

	*C*_11_	*C*_22_	*C*_33_	*C*_44_	*C*_55_	*C*_66_	*C*_12_	*C*_13_	*C*_23_	B
*K*_4_	223.6^a^	—	—	34.6^a^	—	—	82.4^a^	—	—	129.5^a^
	232.2^b^	—	—	31.7^b^	—	—	39.3^b^	—	—	103.6^b^
*A*17	43.8^a^	188.4^a^	16.0^a^	9.8^a^	2.9^a^	58.5^a^	34.5^a^	−1.6^a^	−4.6^a^	10.9^a^
	62.8^b^	199.8^b^	83.2^b^	29.8^b^	9.6^b^	80.6^b^	43.8^b^	1.6^b^	9.3^b^	36.2^b^

^a^Our calculated results at the PBE level.

^b^Our calculated results at the GGA-D2 level.
