# Supplementary material for: Phosphorus K4 Crystal: A New Stable Allotrope
Source: Sci Rep. 2016 Nov 18;6:37528. doi: 10.1038/srep37528 (PMC5114607; doi:10.1038/srep37528)
Supplement: Supplementary Information [file srep37528-s1.docx]

***Supplementary Information for***

**Phosphorus *K*_4_ Crystal: A New Stable Allotrope**

Jie Liu^1,2^ _,_ Shunhong Zhang^1,2^, Yaguang Guo^1,2^, and Qian Wang^* 1,2^

^1^ Center for Applied Physics and Technology, College of Engineering, Peking University; Key Laboratory of High Energy Density Physics Simulation, Ministry of Education, Beijing 100871, China

^2^ Department of Materials Science and Engineering, College of Engineering, Peking University, Beijing 100871, China

*Email: qianwang2@pku.edu.cn

**Figure S1.** **The atomic configurations**. (a) and (b) *K*_4_ phosphorus, and (c) and (d) *A*17 at the end of AIMD simulations for 8 *ps* at 1000 K and 1200 K, respectively. Large supercells of 3×3×3 and 4×4×2 are used for the simulations of *K*_4_ phosphorus and *A*17, respectively.
